# Measuring Transformational Leadership in Establishing Nursing Care Excellence

**DOI:** 10.3390/healthcare7040132

**Published:** 2019-11-04

**Authors:** Sarah E. Moon, Pieter J. Van Dam, Alex Kitsos

**Affiliations:** 1 Australian Institute of Health Service Management, University of Tasmania, Hobart TAS 7005, Australia; 2 School of Medicine, College of Health and Medicine, University of Tasmania, Hobart TAS 7005, Australia; pieter.vandam@utas.edu.au; 3 Wicking Dementia Research & Education Centre, University of Tasmania, Hobart TAS 7005, Australia; alex.kitsos@utas.edu.au

**Keywords:** transformational leadership, Magnet, Multifactor Leadership Questionnaire, nursing workforce, evidence based, healthcare management, leadership development

## Abstract

Transformational leadership (TL) is known to be essential to achieving Magnet^®^ recognition, an internationally prestigious status for nursing care excellence. Since its inception in the 1980s, empirical studies have identified benefits of implementing the Magnet^®^ Model involving improved patient care and nursing workforce outcomes. However, little is known about the leadership styles of nurse managers (NMs) working in a regional Australian context, which may hinder achieving Magnet^®^ status. To close the knowledge gap, a self-administered survey was conducted to measure leadership styles of NMs at a large health organization comprising hospitals with a wide range of service profiles in regional Australia using a validated tool—the Multifactor Leadership Questionnaire (MLQ-6S). One-way of variance (ANOVA) was used to identify statistical significance between respondents’ demographic characteristics (e.g., age, education, gender) and their MLQ-6S scores. Respondents (*n* = 78) reported their leadership styles as more transformational, compared to transactional or passive/avoidant leadership styles. The findings indicated that NMs’ higher education (*p* = 0.02) and older age (*p* = 0.03) were associated with TL styles, whereas passive/avoidant leadership was generally reported by female (*p* = 0.04) and younger (*p* = 0.06) respondents. This study has identified differences in reported leadership styles among NMs, providing a unique organizational insight into developing strategies to improve NMs’ TL, which could help to facilitate the implementation of the Magnet^®^ framework. Healthcare organizations in similar settings could benefit from replicating this study to identify a dominant leadership style and customize strategies to improve TL.

## 1. Introduction

**Figure 1:**
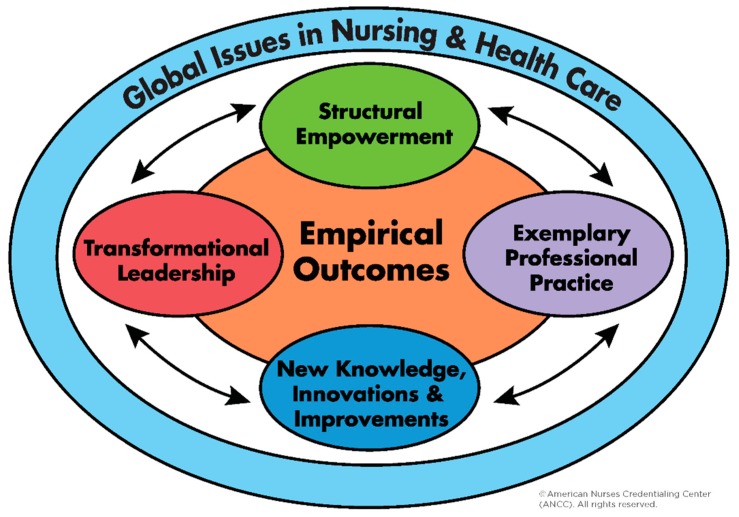
The Magnet^®^ framework.

Transformational Leadership (TL) has been identified as one of the most effective leadership styles in health services [1,2]. In TL, a leader mobilizes followers’ motivations toward an organizational vision by: empowering staff; challenging them beyond the status quo; and recognizing their individual needs and inspirations [3,4]. This approach has resulted in positive organizational performance, such as improved nurse retention and care outcomes [5,6,7,8,9,10,11,12]. The impact of TL is recognized by the American Nurses Credentialing Center (ANCC) through the Magnet Recognition Program^®^ (Magnet^®^) [13], which was developed in the 1980s upon empirical evidence to enable superior nurse recruitment and retention in efforts to combat a national nurse shortage [14,15]. TL is the first component of the Magnet^®^ framework and overarches the other four elements: Structural Empowerment, Exemplary Professional Practice, New Knowledge, Innovations & Improvements, and Empirical Outcomes (Figure 1). To obtain Magnet^®^ recognition, a healthcare facility is required to demonstrate nursing excellence aligned with the framework, evidenced by empirical outcomes of nursing staff and patient care at the organization [13]. 

Recognizing the benefits of Magnet^®^, a healthcare service organization in regional Australia (hereafter the Studied Organization) has embarked on a journey to the Magnet^®^ designation. However, there has been little research examining TL among nurses and midwives in regional Australia, which could provide guidance and support successful Magnet^®^ implementation. Previous Australian studies [16,17,18] conducted Magnet^®^—related surveys to metropolitan settings. Survey instruments used in these studies involved leadership aspects, but lacked a focus on TL, which is the leadership style required to achieve Magnet^®^ recognition. To close the knowledge gap, a survey was conducted to measure leadership styles of the Studied Organization’s NMs.

### 1.1. Transformational Leadership

TL was originally conceptualized in the 1970s by Burns [19] who juxtaposed TL with a comparable leadership style, known as transactional leadership (TAL). Burns defined TL as a relationship in which “leaders and followers raise one another to higher levels of motivation and morality” [19] (p. 6). Conversely, TAL is defined as a relationship, motivated by self-interest rather than a collective good, where a leader exchanges a reward or reinforcement with task accomplishment or failure, respectively [19]. Later, TL was expanded and refined by Bass and Avolio [20] into four distinctive subthemes: Idealized Influence, Inspirational Motivation, Intellectual Stimulation, and Individualized Consideration. A transformational leader builds trust and acts with integrity (Idealized Influence); creates a shared vision and motivates others toward it (Inspirational Motivation); looks for new ideas and beyond the status quo (Intellectual Stimulation); and treats and values others as an individual (Individualized Consideration). In addition, expanding from TL and TAL, Bass and Avolio [20] incorporated another dimension, called passive/avoidant leadership styles, in which a leader fails to action until task failure (Management-B-y-Exception) and is absent from employee-related issues (Laissez-Faire). Together, the elements comprise the Full Range Leadership [4] (Table 1).

Organizational outcomes from TL style have been widely researched and reported across a variety of disciplines [4]. In a nursing context, a systematic review reported that leadership styles which concentrate on people and relationships, such as TL, were associated with superior job satisfaction compared to those task-based styles, including TAL [21]. Demonstrated organizational benefits from TL include better practice environment [7,8,22,23] and superior nurse retention [12,24,25,26].

### 1.2. Transformational Leadership in Magnet^®^

TL establishes a leadership approach to cultivate an environment in which nurse leaders create a vision and support other nurses to lead change [13]. The current Magnet^®^ framework requires the highest responsible nursing officer of an organization to demonstrate TL by fulfilling relevant requirements to qualify as a Magnet^®^ facility [27]. Recent studies have demonstrated that TL styles among nurse managers (NMs) play a key role in developing and sustaining a Magnet^®^ culture [7,28]. Similarly, the role of NMs employed at various levels in creating a positive workplace culture has been consistently highlighted in the Australian literature [29,30,31,32]. Therefore, the imperative of TL can be extended to not only the most senior nursing officer, but also all nurses whose leadership capability impacts on shaping and sustaining Magnet^®^ culture, particularly middle and senior managers.

### 1.3. Magnet^®^ in Regional Australia

Difficulties in recruiting and retaining nurses and midwives are more pronounced in regional Australia [33]. The island state of Tasmania, where the Studied Organization is located, is classified as regional and remote [34] with a population, approximately, of 530,000 [35], with a heightened healthcare demand according to the latest report [36]. Healthcare organizations in the state are increasingly experiencing difficulties in maintaining a sustainable supply of nurses [37]. The Studied Organization is one of few Australian regional health organizations to have officially announced its intention to apply for Magnet^®^ designation. Therefore, local knowledge of leadership styles could assist the Studied Organization to identify strengths and development needs to build a workplace environment that supports Magnet^®^ implementation. 

### 1.4. Study Purpose

This study aimed to measure the leadership styles held by the NMs working in a healthcare organization in regional Australia using a survey tool (MLQ-6S) and to compare the survey result against the first element of the Magnet^®^ framework—Transformational Leadership—in order to inform suitable strategies for leadership development by identifying the unique contextual strengths and improvement opportunities.

## 2. Materials and Methods 

A survey was used to measure prevalent leadership styles among a cohort of NMs. The Multifactor Leadership Questionnaire Form 6S (MLQ-6S) [38], as dependent variables, and additional demographic questions developed by the authors, as independent variables, comprised the study’s survey questionnaire. The MLQ was developed by Bass and Avolio based on research across multiple disciplines, in which its validity and reliability was demonstrated [4,39,40,41,42]. Twenty-one items are included in MLQ-6S to measure three different leadership styles—TL, TAL (Contingent Reward) and passive/avoidant leadership (Passive Management-By-Exception, Laissez-Faire)—under seven dimensions. Each item measures one of the three leadership styles, using a 5-point Likert scale (0 = Not at all through to 4 = Frequently, if not always). The scores from the three items, under the seven subthemes, are then combined to be categorized in High (9–12), Moderate (5–8) and Low (1–4) [38]. Cronbach’s alpha performed for the 21 items of the MLQ-6S were 0.78, indicating acceptable reliability. In addition, seven demographic questions, such as age, gender and education level, were added to the MLQ-6S. 

A convenience sampling identified 183 suitable NMs from the studies organization, ranging from community to acute hospitals. A list of the 183 invitees were obtained from staff directory that was available publicly. Those who were acting in a management role or were employed on a temporary or short-term basis were excluded from the sample. Situated in a hierarchical structure of the organization, this group included middle and senior nurse managers, whose roles incorporated responsibilities and accountabilities for managing and leading nurses and midwives in a unit/ward, a service stream or the organization toward optimized care outcomes. 

The survey was self-administered on an anonymous and voluntary basis via an online survey website, LimeSurvey [43]. The identified NMMs were invited via an email which included survey information and a link to the survey website, providing study information and a consent button. Three weekly follow-up reminders in total were emailed until the survey was closed. Collected over four weeks in January and February 2018, all responses were de-identified, with results aggregated, to avoid potential re-identification by chance. 

All data manipulation and analysis was completed in the statistical computer program, R [44]. One-way ANOVA was used to compare mean MLQ-6S scores between demographic groups (i.e., genders, age-groups, education levels, etc.) and to identify statistically significant differences between the means of two or more independent groups. Results were considered significant when *p* < 0.05, which reflects an association that has unlikely occurred due to chance or error. 

This study was approved by the Tasmanian Health and Medical Human Research Ethics Committee (H0016980).

## 3. Results

A total of seventy-eight participants (*n* = 78) completed the survey with a 42.6% response rate. Incomplete responses were excluded from this study. Sixty-seven (86%) of the respondents were female. The largest age group was 56–60 years (*n* = 21, 27%), with 46% of the participants over 51 years of age. Mid-level nurse managers comprised 73% (*n* = 57) of the respondents. The majority of the NMs (*n* = 61, 78%) had completed post-graduate qualifications (Table 2).

The MLQ scores by the participants indicated that the NMs at the studied organization evaluated their leadership styles more towards TL. Out of the score 12, which is the highest level of the corresponding leadership style, the NMMs scored, on average: 9.0 (High) in TL; 7.2 (Moderate) in TAL; and 6.3 (Moderate) in passive/avoidant leadership. For the TL elements in particular, scores in Individualized Consideration (9.5) and Idealized Influence (9.2) were higher than those in Inspirational Motivation (8.6) and Intellectual Stimulation (8.6). 

Significant associations were seen between NMs’ education, age and gender, and their reported leadership styles. A strong association (*p* = 0.02) was found between NMs’ education and differences in the *Intellectual Stimulation* scores, which overall increased with a higher qualification. NMs’ age revealed statistically significant differences in *Individualized Consideration* (*p* = 0.03), in which scores gradually increased until the peak at age 51–55 years before reducing slightly thereafter. A weaker association (*p* = 0.06) was found between age and *Management-By-Exception* (Passive) where the score peaked at the youngest group, aged 31–35 years. Female respondents reported higher level of *Management-By-Exception* (Passive) with statistical significance (*p* = 0.04). Other variables, including work roles, regions, the length of employment, and the work setting, did not yield statistical significance in associations with MLQ scores (Table 3).

## 4. Discussion

Survey findings indicated that (1) TL was the major leadership styles (TL: 9.0; TAL: 7.2; Passive/avoidant: 6.3) among the NMs of the studied organization; however, (2) the frequency of TL being practiced (‘3 = Sometimes’) could be improved. The high score in TL can be interpreted as a positive result, based on the current evidence that TL has been linked to improved outcomes in nursing workforce and service delivery outcomes in healthcare organizations [7,8,12,21,22,23,24,25,26]. This finding also supports the studied organization’s journey to Magnet^®^ as its measured dominant leadership style aligns with TL. the highest score obtained in *Idealized Influence* (9.2) is noteworthy as Bass and Avolio [4] highlights it as the most potent element in TL. 

However, a caution needs to be applied in that self-identification by the NMs may not necessarily means that these behaviors are displayed. The enactment of leadership is influenced by the context in which leadership is practiced [45]. A work environment might contain barriers to enact TL in day-to-day operations, such as a lack of support from management and clinical staff, lack of authority, and lack of leadership skill development and education [46]. From a Magnet^®^ perspective, a supportive work environment is an important enabler for TL to flourish [47]. For instance, a display of trust and acting with integrity, which are known qualities of *Idealized Influence,* are strong enablers of creating a positive work environment in which Magnet^®^ can be founded upon [48].

In assistance with the improvement in TL at the studied organization, the locally gained knowledge could be considered in strategizing contextual TL development initiatives. These should be suitable for NMs at the studied organization and the initiatives should be accompanied by organization-wide support. 

### 4.1. Intellectual Stimulation

The positive relationship between higher education and Intellectual Stimulation with strong statistical significance (*p* = 0.02) indicates that academic education may improve NMs’ ability to practice stronger TL [49]. Recognizing the importance of education, Magnet^®^ requires that all nurse managers possess a university-level nursing degree and that the highest responsible nursing officer holds at least a master-level qualification [50]. However, the survey findings have indicated an 8% gap in the bachelor’s-degree requirement and a potential cohort (14% holding bachelor’s degree) for a post-graduate qualification uptake. Therefore, the studied organization may benefit from nursing staff undertaking further academic qualifications through a partnership with a local University [51]. To encourage the undertaking, organizational incentive programs, such as career advancement opportunities upon qualification, may be used for current and/or prospect NMs. 

### 4.2. Individualized Consideration

This survey has revealed progressed ageing of the NMs at the studied organization, with 46% of the respondents being aged 51 years or older. This is consistent with the recent nursing workforce sustainability data among Australian hospitals [52] which reported that Tasmania had the biggest group of older nurses and midwives in the nation. Older respondents, however, showed a statistically stronger (*p* = 0.03) association with an increased level of *Individualized Consideration*, while the youngest group scored the highest in *Management-By-Exception* (Passive) with a weaker significance (*p* = 0.06). Passive/avoidant leadership styles, including *Management-By-Exception* (Passive), have been linked to negative impacts on nursing workforce [7,53]. Combining the two findings, it can be argued that older NMs at the studied organization may possess more qualities of TL than their younger counterparts, which is consistent with other studies that have linked older age to a higher level of TL [49,54]. From a workforce sustainability standpoint, the progressed ageing of the nurse managers NMs at the studied organization could be capitalized by potentially using these nurses and midwifes as mentors or coaches. *Individualized Consideration* has been positively associated with change implementation in healthcare organizations through relational and individualized measures, such as mentoring and coaching [55,56,57]. Thus, the identified strengths in *Individualized Consideration* in older NMs of the studied organization could be expanded and transferred to other generations, particularly the young, by developing and using a formal mentoring and coaching program. Accordingly, this may assist Magnet^®^ implementation at the studied organization. 

### 4.3. Management-By-Exception 

Interestingly, the statistically significant (*p* = 0.04) association between the participants’ gender and *Management-By-Exception* (Passive) obtained in this survey appears to contradict current literature in gender and leadership. In this study, the female NMs reported higher level of *Management-By-Exception* (Passive). However, previous studies [58,59] confirmed that female groups tend to show TL styles more often. The discrepancy may perhaps be attributed to the over-representation of female gender (86%) in a small sample (*n* = 78) in this study. Further investigation would be required to determine the validity of the gender difference in TL among the NMs at the Studied Organization. 

### 4.4. Limitations and Future Research

Although inherent limitations exist due to the nature of a quantitative research design used in this study, this study has contributed to advancing nursing leadership development, particularly from a Magnet^®^ perspective. The quantitative method limited opportunities to unveil details beyond the survey items. The details may have led to specific and unique factors to optimize TL at the studied organization and potentially in the regional Australian contexts. The self-report approach may have induced over—or underestimation in leadership styles. In addition, the small sample (*n* = 78) may have limited the generalizability of the results in a broader regional Australian context. Lastly, with regard to the imbalance in the sample size between male and female participants addressed above, a more balanced sex proportion may improve the representation of potential differences in leadership behaviors affected by a person’s sex. On the other hand, this study has provided a foundation for the nursing leadership evaluation within the context of regional healthcare organization in Australia in the context of Magnet^®^ recognition. Further replication and/or regular evaluations may be useful to help inform leadership development aligned to Magnet^®^ recognition at the healthcare organizations in similar contexts. Additionally, local impacts of TL on nursing and care outcomes within the Studied Organization could be explored to further promote TL development.

## 5. Conclusions

This study identified potential enablers of TL development towards Magnet^®^ recognition in a regional Australian context. The findings indicated specific TL components (*Individualized Consideration* and *Intellectual Stimulation*) which could be improved by leveraging the maturity of current and/or prospect NMs and advancing their academic education, respectively. Current evidence, as discussed above, supports optimized TL assists healthcare organizations with achieving better care outcomes, celebrated by obtaining Magnet^®^ designation, and retaining the sustainability of nursing and midwifery workforce. This study provides decision-makers of regional healthcare organizations with a unique insight to help determine priorities toward TL development and achieve improved organization outcomes.

## Figures and Tables

**Table 1:** Elements of Full Range Leadership [4].

Full Range Leadership	Element
Transformational leadership	Idealized Influence
	Inspirational Motivation
	Intellectual Stimulation
	Individualized Consideration
Transactional leadership	Contingent Rewards
	Active Management-By-Exception
Passive/avoidant leadership	Passive Management-By-Exception
	Laissez-Faire

**Table 2:** Demographic responses of survey participants.

Category	Description	No. of Responses (%)
Total		78 (100)
Gender	Female	67 (86)
	Male	11 (14)
	Other	0
Age	31–35 years	4 (5)
	36–40 years	7 (9)
	41–45 years	6 (7)
	46–50 years	15 (19)
	51–55 years	17 (22)
	56–60 years	21 (27)
	61 or older	8 (10)
Region	North	27 (35)
	North West	19 (24)
	South	32 (41)
Role	Nurse Manager with no clinical responsibilities	10 (13)
	Nurse (Unit) Manager	47 (60)
	Assistant Director of Nursing	12 (15)
	(Co-) Director or Executive Director of Nursing	9 (12)
Highest education	Hospital/training Certificate	6 (8)
	Bachelor’s Degree	11 (14)
	Post-graduate Certificate	12 (15)
	Post-graduate Diploma	22 (28)
	Master’s Degree	27 (35)
	Doctoral Degree	0 (0)
Work length at current hospital	Less than 1 year	1 (1)
	1–5 years	7 (9)
	6–10 years	12 (15)
	11–15 years	8 (10)
	16–20 years	8 (10)
	21 years plus	42 (54)
Work setting	Community/Primary care	4 (5)
	Hospital/Acute care	66 (85)
	Non-hospital inpatient facility (sub-acute care)	3 (4)
	Other	5 (6)

**Table 3:** Analysis of MLQ-6S scores by respondent demographics.

MLQ Factors (MLQ-6S)		Factor 1	Factor 2	Factor 3	Factor 4	Factor 5	Factor 6	Factor 7
Leadership styles		Transformational				Transactional	Passive/Avoidant	
MLQ-6S score average		9.2	8.6	8.7	9.5	7.2	8.4	4.1
Average score for leadership style		9.0				7.2	6.3	
Demographic variance		Idealized Influence (mean (SD ^1^))	Inspirational Motivation (mean (SD))	Intellectual Stimulation (mean (SD))	Individualized Consideration (mean (SD))	Contingent Reward (mean (SD))	Management-By-Exception (mean (SD))	Laissez Faire (mean (SD))
Gender	Female	9.2 (1.4)	8.6 (1.5)	8.6 (1.7)	9.5 (1.5)	7.2 (2.3)	8.6 (1.5)	4.3 (2.1)
	Male	9.1 (1.6)	8.8 (1.1)	8.7 (1.4)	9.1 (1.3)	7.0 (2.0)	7.5 (2.1)	3.2 (1.8)
*p*-value ^2^		0.79	0.66	0.88	0.37	0.74	0.04	0.07
Age	31–35	9.5 (0.6)	8.8 (1.3)	9.3 (2.5)	8.5 (1.3)	5.8 (1.3)	9.3 (1.7)	4.5 (2.1)
	36–40	9.0 (1.5)	9.4 (1.0)	8.9 (1.6)	8.6 (1.5)	7.7 (2.0)	7.1 (0.9)	3.6 (2.2)
	41–45	10.0 (1.4)	7.8 (1.6)	7.5 (0.8)	8.8 (1.5)	6.7 (2.1)	7.7 (1.0)	2.8 (2.4)
	46–50	9.2 (1.5)	8.9 (1.4)	8.8 (1.7)	8.9 (1.2)	7.1 (2.6)	8.3 (1.2)	4.1 (1.8)
	51–55	9.5 (1.5)	9.1 (1.4)	9.2 (1.4)	10.2 (1.7)	7.9 (2.3)	8.4 (2.2)	4.2 (2.3)
	56–60	8.8 (1.3)	8.2 (1.5)	8.4 (1.7)	9.8 (1.2)	7.1 (1.9)	9.1 (1.5)	4.8 (2.0)
	≥ 61	9.0 (1.2)	8.0 (1.1)	8.1 (2.2)	9.8 (1.2)	7.0 (2.8)	7.9 (1.6)	3.6 (1.6)
*p*-value		0.48	0.13	0.34	0.03	0.66	0.06	0.46
Highest completed Education	Hospital training	8.8 (1.2)	7.2 (1.3)	8.0 (1.4)	9.2 (1.2)	7.2 (1.8)	9.3 (1.6)	4.7 (0.8)
	Bachelor’s	9.1 (1.0)	8.9 (0.9)	8.6 (1.2)	9.0 (1.5)	7.3 (2.3)	8.2 (1.0)	4.6 (1.5)
	Post-grad Certificate	9.0 (1.3)	8.4 (1.2)	7.4 (1.4)	9.5 (1.3)	6.6 (2.4)	8.3 (1.5)	4.3 (1.6)
	Post-grad Diploma	9.6 (1.4)	8.5 (1.6)	8.7 (1.6)	9.7 (1.6)	7.4 (2.4)	8.6 (1.7)	4.3 (2.4)
	Master’s	9.1 (1.6)	8.9 (1.5)	9.3 (1.7)	9.5 (1.5)	7.3 (2.2)	8.2 (1.9)	3.6 (2.3)
	Doctoral	0	0	0	0	0	0	0
*p*-value		0.70	0.08	0.02	0.73	0.89	0.51	0.62

^1^ SD: standard deviation; ^2^ *p*-value is significant at 0.05.
